# Role of Tacrolimus C/D Ratio in the First Year After Pediatric Liver Transplantation

**DOI:** 10.3389/fped.2021.659608

**Published:** 2021-06-02

**Authors:** Benas Prusinskas, Sinja Ohlsson, Simone Kathemann, Denisa Pilic, Kristina Kampmann, Rainer Büscher, Andreas Paul, Lars Pape, Peter F. Hoyer, Elke Lainka

**Affiliations:** ^1^Department of Pediatrics II, Pediatric Gastroenterology, Hepatology and Liver Transplantation, University Children's Hospital, Essen, Germany; ^2^Department of Pediatrics II, Pediatric Nephrology and Kidney Transplantation, University Children's Hospital Essen, Essen, Germany; ^3^Department of General, Visceral, and Transplantation Surgery, University Medicine Essen, Essen, Germany

**Keywords:** tacrolimus, calcineurin inhibitor, pediatric liver transplantation, nephrotoxicity, metabolism

## Abstract

**Background:** The calcineurin inhibitor (CNI) tacrolimus (TAC) is a cornerstone agent in immunosuppressive therapy in pediatric liver transplantation (LTX). Adverse effects limit the use of CNI. In adults, calculating the individual TAC metabolism rate allows to estimate the transplant recipient's risk for therapy-associated complications.

**Methods:** A retrospective, descriptive data analysis was performed in children who had undergone LTX in 2009–2017 and had received TAC twice daily in the first year after LTX. A weight-adjusted concentration/dose ratio (C/D ratio) was calculated [TAC trough level/(daily TAC dose/body weight)] every 3 months after LTX to estimate the average individual TAC metabolism rate. Depending on the C/D ratio, all patients were divided into two groups: fast metabolizers (FM) and slow metabolizers (SM). Clinical and laboratory parameters were analyzed as risk factors in both groups.

**Results:** A total of 78 children (w 34, m 44, median age at LTX 2.4; 0.4–17.0 years) were enrolled in the study. FM (SM) had a mean C/D ratio of <51.83 (≥51.83) ng/ml/(mg/kg). FM were younger at the time of LTX (median age 1.7; 0.4–15.8 years) than SM (5.1, 0.4–17.0), *p* = 0.008. FM were more likely to have biliary atresia (20/39, 51%) compared to SM (11/39, 28%), *p* = 0.038, whereas SM were more likely to have progressive familial intrahepatic cholestasis (9/39, 23%) vs. in FM (1/39, 3%), *p* = 0.014. Epstein–Barr virus (EBV) infection occurred more frequently in FM (27/39, 69%) than SM (13/39, 33%), *p* = 0.002. Three FM developed post-transplant lymphoproliferative disorder. The annual change of renal function did not differ in both groups (slope FM 1.2 ± 0.6; SM 1.4 ± 0.8 ml/min/1.73 m^2^ per year, and *p* = 0.841).

**Conclusions:** Calculation of individual, weight-adjusted TAC C/D ratio is a simple, effective, and cost-efficient tool for physicians to estimate the risk of therapy-associated complications and to initiate individual preventive adjustments after pediatric LTX. Lower TAC levels are tolerable in FM, especially in the presence of EBV infection, reduced renal function, or when receiving a liver transplant in the first 2 years of life.

## Introduction

Over the last decades, liver transplantation (LTX) has become a safe treatment method with excellent results and low mortality for children with end-stage liver disease (1). Calcineurin inhibitors (CNI) are the mainstay of immunosuppressive therapies after pediatric LTX (2). The side effects of tacrolimus (TAC) are similar in adults and children. However, children have a higher therapy-related risk for Epstein–Barr virus (EBV) infection and post-transplant lymphoproliferative disorder (PTLD) because children are more likely to be EBV naïve at the time of LTX (3, 4). Acute and chronic CNI nephrotoxicity is defined by clinical (impairment of renal function, arterial hypertension), laboratory (e.g., acid–base disorders, electrolyte disorders), and histological findings (5, 6). Acute CNI nephrotoxicity is an important cause for morbidity after LTX; it usually develops in the early stages after transplantation, when high CNI doses are required and mostly reversible (6, 7).

One of the biggest challenges of the immunosuppressive therapy with TAC is finding the right balance between efficacy (avoiding a graft rejection) and toxicity (especially nephrotoxicity) (6). Many studies have focused on strategies to minimize or avoid CNI nephrotoxicity, including dose reduction, switch of therapy, or complete avoidance of CNIs. These methods are effective in reducing nephrotoxicity; however, the standard of care remains unchanged because the immunosuppressive efficacy of TAC is superior to any other strategy (6, 8–10).

In recent years, predictive markers which could help to estimate an individual TAC metabolism and risk for toxicity were evaluated. A promising method is the calculation of the TAC metabolism rate, defined by the trough TAC blood concentration, normalized by its daily dose. The individual rate of TAC metabolism is measured in order to estimate individual TAC exposure (11–13). It has been shown that this simple and low-cost yet effective tool could help physicians not only to predict the individual risk for TAC toxicity but also to predict the graft function, risk for graft rejection, or even overall patient survival (11). Patients after liver or kidney transplantation who had a fast TAC metabolism rate had a higher risk of TAC-associated kidney disease compared to those with a low metabolism rate (14, 15). Unlike adult patients, children's body weights vary according to their age. Subsequently, there are considerable variations in the total daily TAC dose in the different weight classes. The proposed formula for the calculation of the concentration/dose ratio (C/D ratio) in children is difficult without adjustment for body weight. Moreover, the pharmacokinetics of TAC (intestinal resorption, distribution, and elimination) in children have a greater variability compared to adult patients (16). It is unclear whether the calculation of the C/D ratio in pediatric patients could be used as a predictor in the same way as in adult patients.

The aim of this study was to evaluate whether there is an association between the weight-adjusted TAC C/D ratio and adverse effects of TAC in pediatric patients. Furthermore, we analyzed predictive factors, which could influence the weight-adjusted C/D ratio.

## Materials and Methods

### Patients

**Figure 1:**
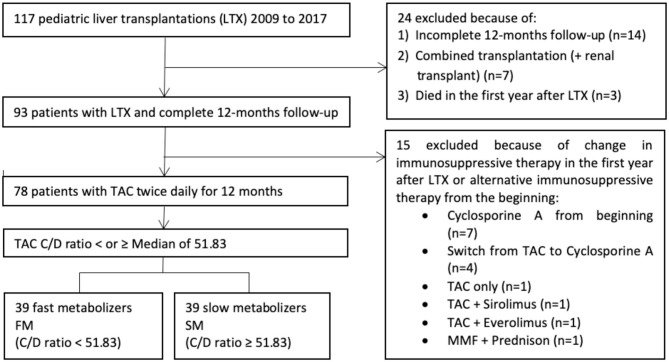
Enrolment in the study.

In this retrospective study, we included all pediatric patient, who had undergone LTX at University Children's Hospital Essen from 2009 until 2017, according to defined inclusion criteria (Figure 1).

### Immunosuppression in the First Year After LTX

Every patient had received a combined immunosuppressive therapy with TAC, mycophenolate mofetil (MMF), and corticosteroids following LTX. Corticosteroids (prednisolone or prednisone) had been started with a single dose of 300 mg/m^2^ intravenous administration during LTX, followed by oral or intravenous administrations at 15 mg/m^2^ once daily, followed by slowly reducing the doses. The starting dose of TAC was 0.05–0.1 mg/kg twice daily. MMF was started at day 3 after LTX with a dose of 300 mg/m^2^ twice daily. The dose was increased to 600 mg/m^2^ twice daily on day 5. In the further course, doses were adjusted following the results of therapeutic drug monitoring.

### Clinical Data

The demographic and clinical patient characteristics have been collected at the time of LTX, at study enrolment, and were longitudinally documented during follow-up visits. Those included date of birth, sex, indication, and date of LTX, graft type (whole liver/split liver), type of LTX (living/deceased donor, LD/DD), and type of surgical connection of bile ducts [direct anastomosis/biliodigestive anastomosis (BDA)]. In a few cases, a primary direct anastomosis of bile ducts had to be surgically revised within the first 3 months after LTX. In all these cases, a secondary BDA was done. These patients were classified as patients with BDA. Body weight and height, as well as calculated anthropometric parameters [body mass index (BMI) and body surface area (BSA)], were obtained at the time of LTX and every 3 months after LTX.

The following laboratory parameters were obtained at the time of LTX as well as 3, 6, 9, and 12 months after LTX: alanine aminotransferase (ALT), total bilirubin (Bili), quick value, red blood cell count (RBC), magnesium (Mg), albumin (Alb), immunoglobulin G (IgG), urea (Urea), and creatinine. Screening for EBV infection was performed in blood samples using polymerase chain reaction (PCR). EBV infection was considered positive if the viral deoxyribonuclei c acid (DNA) load in the blood was >3,000 copies/ml (test method until 2016) or >150 IU/ml (test method after 2016).

### TAC C/D Ratio

A weight-adjusted tacrolimus C/D ratio was calculated and used as a surrogate marker for individual TAC metabolism rate. The following formula was used:

$$\begin{array}{l} \text{C}/\text{Dratio}\left(\text{ng}/\text{ml}/\left(\text{mg}/\text{kg}\right)\right)=\frac{\left(\text{TAC through level in blood }\left(\text{ng}/\text{ml}\right)\right)}{\left(\text{daily dose}\left(\text{mg}\right)/\text{body weight }\left(\text{kg}\right)\right)} \end{array}$$

### Definition of Fast and Slow Metabolizers

The C/D ratio was assessed 3, 6, 9, and 12 months after LTX. A mean value from these four measures was calculated in every patient. The median from all of these average values was 51.83 ng/ml × 1/(mg/kg) and has been used to divide all patients in two groups: patients with an average C/D ratio of <51.83 ng/ml/(mg/kg) were classified as fast metabolizers (FM). Patients who had a C/D ratio of ≥51.83 were classified as slow metabolizers (SM).

### Renal Function

An estimated glomerular filtration rate (eGFR) was accurately calculated using creatinine-based Schwartz formula [eGFR in ml/min/1.73 m^2^: height (cm) × *K*/creatinine (mg/dl); *K* = 0.45 within the first year of life, *K* = 0.55 after the first year of life, *K* = 0.7 for pubertal boys] for assessment of renal function (17). The eGFR was calculated 3, 6, 9, and 12 months after LTX. Change of renal function was defined as a change in eGFR during the course of the first year after LTX (slope). Impaired renal function was defined as eGFR <90 ml/min/1.73 m^2^, seen at least once during the first year after LTX (excluding patients who already had impaired eGFR prior to therapy with TAC).

### Data Collection and Statistical Analysis

Retrospective data collection was carried out using the computerized and paper-based databases of the University Children's Hospital Essen. The statistical analysis was performed using Microsoft Excel 2016, version 16.0 (Microsoft, Redmond, Washington, USA) and GraphPad Prism, version 9.00 for MacOS (Graph Pad Software, San Diego, California, USA). Univariate statistical analyses were used to describe demographic and clinical parameters. Linear regression analysis was performed to assess the change in renal function as estimated by the slope for all eGFRs. Chi-square test or Fisher's exact test was used for categorical variables to quantify evidence of differences between the two groups. Shapiro–Wilk test was used to determine the distribution of continuous variables (mean and standard deviation for normal distribution and median and range for non-normal distribution). To compare two groups with continuous variables, two-sample *t*-test (normal distribution) or Mann–Whitney *U*-test (non-normal distribution) was used. The association between two groups of continuous variables was quantified using Spearman's rank correlation coefficient. *P*-values were considered statistically significant if *p* < 0.05.

### Ethics Commission

This study has been approved by the ethics committee and the data protection responsible officer at the University of Duisburg-Essen (vote 19-8902-BO).

## Results

### Patient Cohort

A total of 117 children have received a liver graft in the period from 2009 to 2017. There were 78 patients who met the final inclusion criteria and were thus enrolled in the study (Figure 1). The study population consisted of 44 boys (56.4%) and 34 girls (43.6%). Demographic, anthropometric, and clinical data are represented in Table 1.

**Table 1:** Descriptive demographic, anthropometric, and clinical data of patients (*n* = 78).

	**Median**	**Minimum**	**Maximum**
Age (years)	2.40	0.38	17.04
Body height (cm)	88.00	60.00	173.00
Body weight (kg)	12.85	4.40	65.00
BSA (m^2^)	0.56	0.28	1.73
BMI (kg/m^2^)	16.15	9.85	23.73
Male/female	44 (56%)	34 (44%)	
First/second liver transplantation	65 (83%)	13 (17%)	
Living/deceased donor	23 (29%)	55 (71%)	
Whole/split liver	38 (49%)	40 (51%)	
Biliodigestive/direct anastomosis	51 (65%)	27 (35%)	

### TAC C/D Ratio and Patients' Characteristics

**Figure 2:**
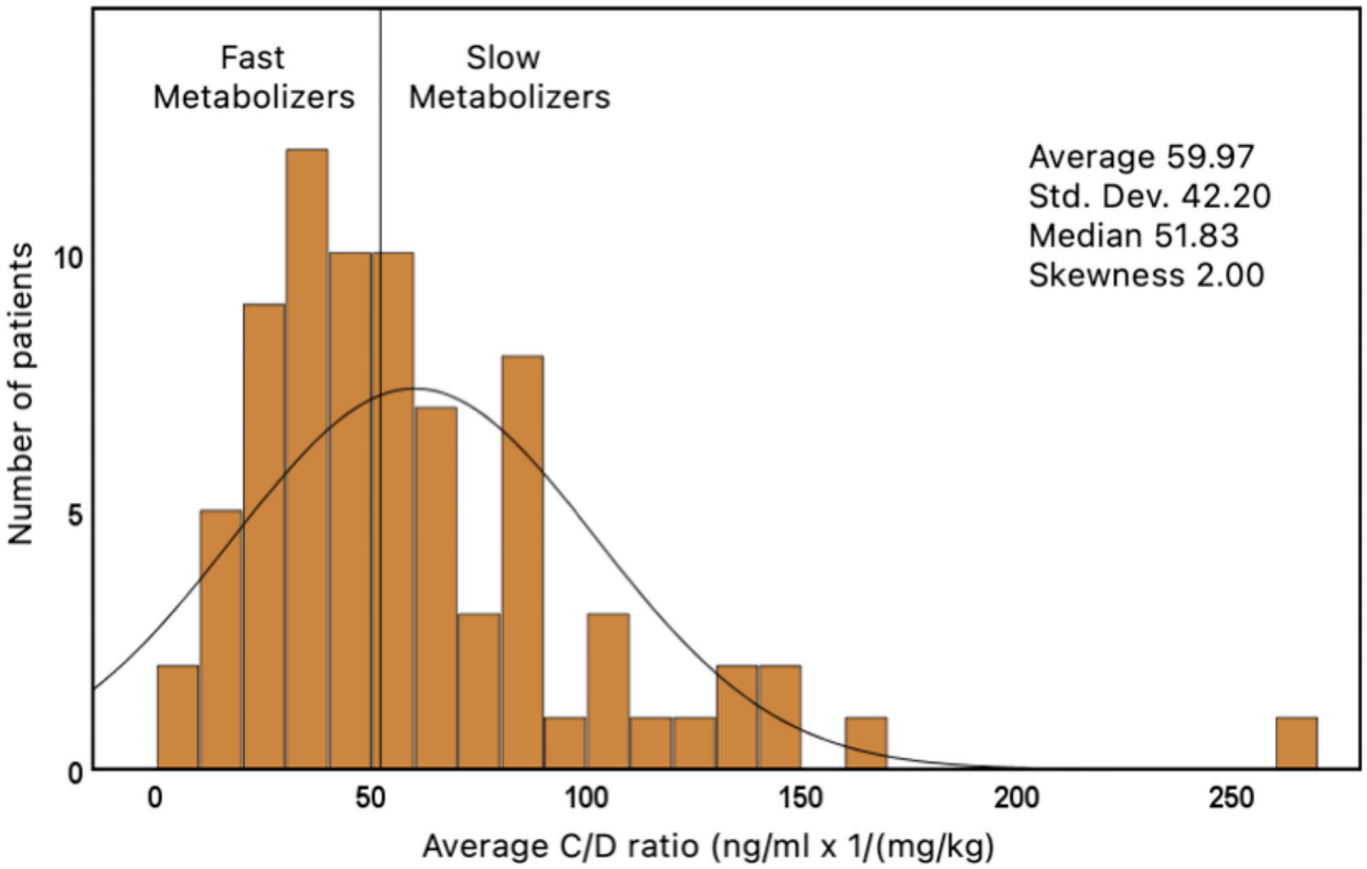
Distribution of tacrolimus concentration/dose ratio (*n* = 78).

The average C/D ratio was used to divide the patients into two groups. The median of all average values was 51.83 ng/ml/(mg/kg) (range: 3.5–265.7). Children who had an average C/D ratio of <51.83 were classified as FM (*n* = 39). Children with a ratio ≥51.83 were classified as SM (*n* = 39) (Figure 2).

**Figure 3:**
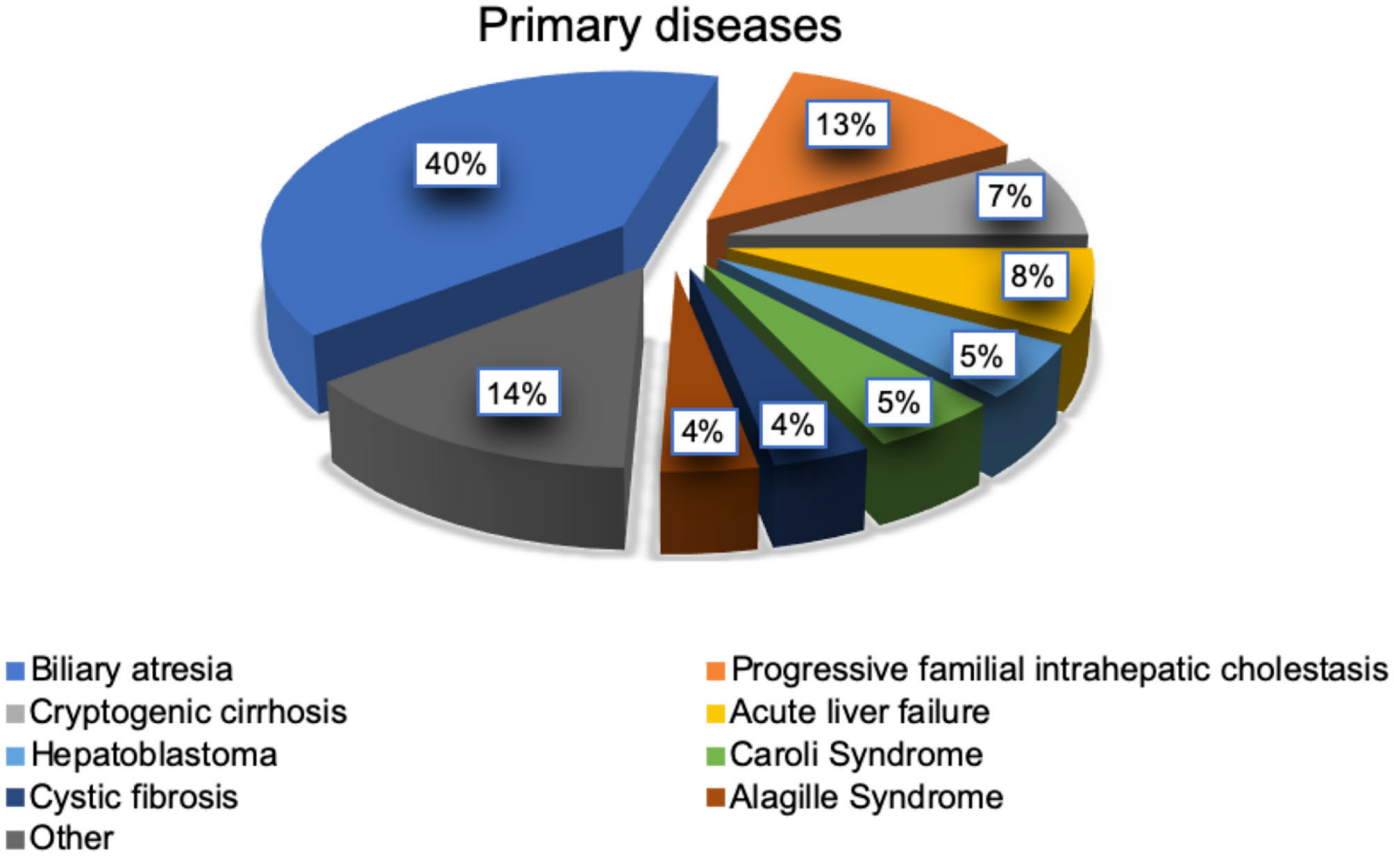
Indications for liver transplantation in study population (*n* = 78).

FM were considerably younger at the time of LTX (median age, 1.7 years) and therefore had a smaller body size and lower body weight than the slow metabolizers (median age 5.1 years), *p* = 0.008 (Table 2). Moreover, we found a significantly weak positive correlation (*r* = 0.246, *p* = 0.03) between patients' age and TAC C/D ratio. The most frequent indication for LTX in both groups was biliary atresia (31/78, 40%; Figure 3). The average age at LTX in children with biliary atresia was 2.34 years (median age, 0.65 years). FM were more likely to have biliary atresia (20/39, 51%) compared to SM (11/39, 28%), *p* = 0.038, while SM were more likely to have progressive familial intrahepatic cholestasis (PFIC) (9/39, 23%) compared to FM (1/39, 3%), *p* = 0.014. The average age of children with PFIC was 8.48 years (median age, 7.84 years). No differences were found in other underlying conditions.

**Table 2:** Clinical characteristics of the patients in both metabolizer groups (*n* = 78).

	**Fast metabolizers, *n* = 39**	**Slow metabolizers, *n* = 39**	***p*-value**
Age (years)	1.7 (0.4–15.8)	5.1 (0.4–17.0)	0.008^a^
Body height (cm)	81.0 (6.0–173.0)	109.0 (61.0–172.0)	0.035^a^
Body weight (kg)	10.4 (4.4–65.0)	20.0 (5.8–57.3)	0.020^a^
BSA (m^2^)	0.5 (0.3–1.7)	0.8 (0.3–1.6)	0.021^a^
BMI (kg/m^2^)	15.6 (11.5–23.7)	16.5 (9.9–22.9)	0.028^a^
Male/female	24 (62%)/15 (39%)	20 (51%)/19 (49%)	0.361^b^
First/second liver transplantation (LTX)	32 (82%)/7 (18%)	33 (84%)/6 (15%)	0.761^b^
Living/deceased donor	11 (18%)/28 (72%)	12 (31%)/27 (69%)	0.804^b^
Whole/split liver	18 (46%)/21 (54%)	20 (51%)/19 (49%)	0.651^b^
Biliodigestive/direct anastomosis	28 (72%)/11 (28%)	23 (59%)/16 (41%)	0.234^b^
MMF withdrawn in first year after LTX (yes/no)	20 (51%)/19 (49%)	20 (51%)/19 (49%)	–

*Age, body height, body weight, BSA, and BMI are illustrated using median value and range (minimum–maximum)*.

a *P-values from Mann–Whitney U-test*.

b *P-values from chi-square test*.

### TAC C/D Ratio and Renal Function

**Figure 4:**
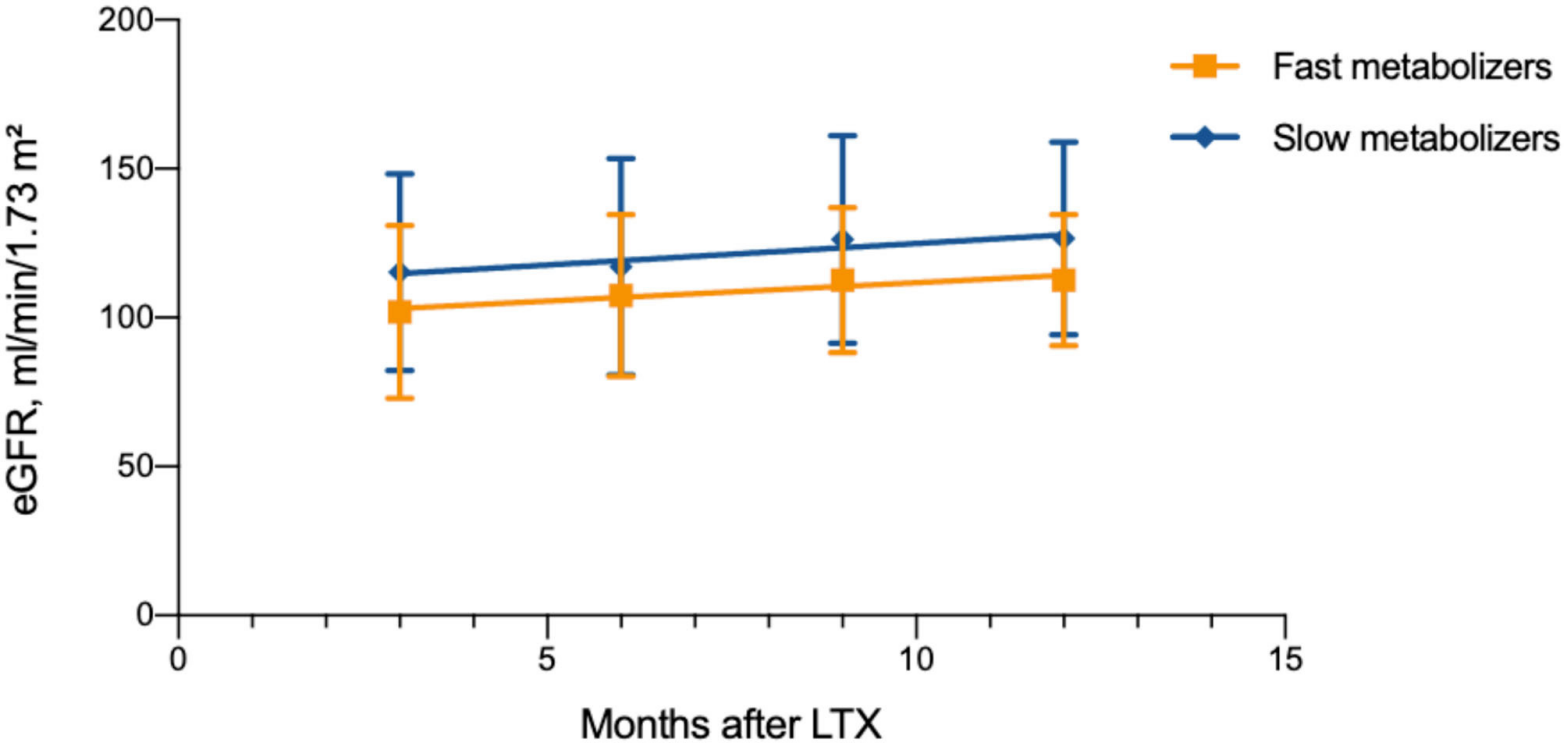
Renal function after liver transplantation in different age and metabolism groups (*n* = 78).

The eGFR (creatinine-based Schwartz formula) was calculated 3, 6, 9, and 12 months after LTX. The mean renal function after LTX was normal: 108.6 ± 5.1 ml/min/1.73 m^2^ in the group of FM and 121.3 ± 6.0 ml/min/1.73 m^2^ in the group SM, *p* = 0.018 (Table 3). The change in renal function was defined using slopes, which showed no significant difference between both groups, as the renal function increased on average in both groups during the first year after LTX (slope FM 1.2 ± 0.6; SM 1.4 ± 0.8 ml/min/1.73 m^2^ per year, *p* = 0.841; Figure 4). During the whole observation period, we identified selective nephrotoxicity with impaired eGFR in 10/30 (33%) FM and 5/32 (16%) SM (*p* = 0.104) during the first 6 months after LTX.

**Table 3:** Various laboratory parameters in both metabolizer groups after liver transplantation (LTX) (*n* = 78).

	**Fast metabolizers**	**Slow metabolizers**	***p*-value**
**ALT (U/L)**			
3 months^a^	28 (10–189)	33 (8–248)	0.099^b^
6 months^a^	26 (8–113)	37 (9–243)	0.100^b^
9 months^a^	27 (1–177)	37 (8–172)	0.060^b^
12 months^a^	31 (5–276)	29 (5–250)	0.764^b^
First year average	29 (13–15)	37 (8–227)	0.032^b^
**Bili (mg/dl)**			
3 months^a^	0.4 (0.1–4.1)	0.4 (0.1–14.6)	0.093^b^
6 months^a^	0.3 (0.1–3.3)	0.5 (0.1–17.3)	0.018^b^
9 months^a^	0.4 (0.1–2.8)	0.5 (0.2–3.8)	0.021^b^
12 months^a^	0.3 (0.1–3.4)	0.6 (0.1–23.7)	0.002^b^
First year average	0.4 (0.1–3.4)	0.6 (0.2–21.6)	0.004^b^
**Quick value (%)**			
3 months^a^	89 ± 13	86 ± 18	0.387^c^
6 months^a^	89 ± 15	86 ± 14	0.266^c^
9 months^a^	88 ± 16	85 ± 17	0.428^c^
12 months^a^	89 ± 17	87 ± 15	0.480^c^
First year average	89 ± 13	86 ± 13	0.268^c^
**Alb (g/dl)**			
3 months^a^	4.2 (3.3–4.8)	4.3 (2.2–5.1)	0.099^b^
6 months^a^	4.2 (3.2–4.7)	4.3 (3–4.7)	0.100^b^
9 months^a^	4.2 (2.9–51)	4.2 (3–4.8)	0.060^b^
12 months^a^	4.3 (3.2–5.5)	4.2 (2.9–4.7)	0.764^b^
First year average	4.13 (3.7–4.7)	4.33 (2.9–4.8)	0.381^b^
**Mg (mmol/L)**			
3 months^a^	0.8 ± 0.1	0.7 ± 0.1	0.296^c^
6 months^a^	0.8 ± 0.1	0.8 ± 0.1	0.640^c^
9 months^a^	0.8 ± 0.1	0.8 ± 0.1	0.958^c^
12 months^a^	0.8 ± 0.1	0.8 ± 0.1	0.966^c^
First year average	0.8 ± 0.1	0.8 ± 0.1	0.700^c^
**RBC (/pl)**			
3 months^a^	4.5 ± 0.7	4.6 ± 0.7	0.641^c^
6 months^a^	4.6 ± 0.8	4.5 ± 0.8	0.957^c^
9 months^a^	4.7 ± 0.6	4.6 ± 0.9	0.460^c^
12 months^a^	4.6 ± 0.6	4.6 ± 0.7	0.857^c^
First year average	4.5 ± 0.7	4.6 ± 0.7	0.701^c^
**IgG (g/dl)**			
3 months^a^	3.9 (1.5–15.1)	6.4 (1.6–18.6)	0.043^b^
6 months^a^	5.7 (1.4–16.7)	6.8 (2.4–18.3)	0.187^b^
9 months^a^	7.0 (1.4–16.5)	7.5 (3.8–20.7)	0.199^b^
12 months^a^	7.8 (1.5–18.6)	7.5 (2.8–22.4)	0.708^b^
First year average	6.4 (3.1–15.4)	7.3 (3.4–18.2)	0.265^b^
**eGFR (bedside Schwartz formula), ml/min/1.73 m**^**2**^			
3 months^a^	102.0 ± 29.0	115.3 ± 33.0	0.063^c^
6 months^a^	107.4 ± 27.3	117.0 ± 36.5	0.190^c^
9 months^a^	112.5 ± 24.5	126.2 ± 34.8	0.049^c^
12 months^a^	112.6 ± 22.0	126.9 ± 32.3	0.027^c^
First year average	108.6 ± 5.1	121.3 ± 6.0	0.018^c^
**Urea, mmol/L**			
3 months^a^	5.0 (2.1–13.0)	5.0 (1.8–10.0)	0.645^b^
6 months^a^	4.6 (1.4–13.5)	4.3 (2.5–17.8)	0.656^b^
9 months^a^	4.3 (1.1. −13.2)	4.6 (2.5–16.0)	0.947^b^
12 months^a^	5.0 (1.4–12.8)	4.6 (2.1–15.0)	0.253^b^
First year average	5.0 (2.4–12.1)	4.5 (2.7–14.7)	0.342^b^
**EBV infection, patients**			
Detected/all patients (%)	27/39 (69%)	13/39 (33%)	0.002^d^

*ALT, Bili, Alb, IgG, and Urea are illustrated using median value and range (minimum–maximum). Quick value, Mg, RBC, and eGFR are illustrated using average value ± standard deviation*.

a *Months after LTX*.

b *P-values from Mann–Whitney U-test*.

c *P-values from T-test*.

d *P-values from chi-square test*.

### TAC C/D Ratio and Liver Function Tests

SM showed higher normalized ALT values in the first year after LTX (median 37 U/L, range 8–227) compared to FM (29 U/L, 13–165, *p* = 0.003). Analysis of values at 3, 6, 9, and 12 months after LTX showed no significant differences. The normalized mean values of Bili at 6, 9, and 12 months after LTX were significantly higher in the group of SM. In the first year after LTX, SM had higher normalized Bili (median 0.6 mg/dl, range 0.2–21.6) than FM (0.4 mg/dl, 0.13–3.4, *p* = 0.004; Table 3). In addition, we found a significant positive correlation between total serum bilirubin and Tac C/D ratio (*r* = 0.376, *p* = 0.001).

### TAC C/D Ratio and Other Laboratory Parameters (Mg, RBC, Alb, and IgG)

FM showed a significantly lower IgG level in the first 3 months after LTX (median 3.9 g/dl, range 1.5–15.1) compared to SM (6.4 g/dl, 1.6–18.6, *p* = 0.043). At this measuring point, 22/36 (61%) FM and 17/38 (45%) SM had age-correlated hypogammaglobulinemia (*p* = 0.159). No significant differences of IgG have been found at 6, 9, and 12 months after LTX. No significant differences have also been found in the RBC count, Mg concentration, and serum albumin between both groups (Table 3). The Mg levels were influenced by Mg substitution in 52% of all children (42% FM and 61% SM, *p* = 0.121).

### TAC C/D Ratio and Complications After LTX (EBV Infection, PTLD, and Graft Rejection)

All patients were screened for EBV infection every 3 months after LTX. Positive EBV viremia was found at least once in the first year after LTX in 40 out of 78 patients (51%). In the group of FM, EBV viremia was found significantly more often (27/39, 69%) than in the group of SM (13/39, 33%), *p* = 0.002 (Table 3). During the first year after LTX, three out of 78 children (4%) developed PTLD. All three children with PTLD were FM, at the time of LTX very young (respectively, 0.57, 0.65, and 0.97 years old) and had an extremely low average C/D ratio [respectively, 3.5, 10.2, and 13.5 ng/ml/(mg/kg)], which shows a high overall TAC exposition. The absolute TAC doses in these children were also high (7.5, 6, and 13 mg/d). All three patients developed PTLD primarily in the gastrointestinal tract. The bile ducts have been connected using roux-Y-anastomose. In addition, all three children had tested positive for EBV infection shortly prior to the diagnosis of PTLD, although the virus count was not very high (respectively, 2217, 3463, and 7411 copies/ml). Additionally, IgG levels were decreased in all three patients at the time of diagnosis (respectively, 3.0, 1.4, and 2.5 g/dl). A histologically proven graft rejection was detected in 27 out of 78 patients (35%). In the first year after LTX, 10/39 (26%) FM and 17/39 (44%) SM have developed graft rejection. There was no statistically significant difference between the groups (*p* = 0.096).

### TAC C/D Ratio and Diarrhea

A detailed clinical history, including diarrhea, was collected every 3 months after LTX. Ten out of 78 (13%) patients were excluded (three because of the development of PTLD and seven because of missing data). Eight of the remaining 68 patients (12%) developed unexplained chronic diarrhea in the first year after LTX (children who had an obvious reason for diarrhea, e.g., viral/bacterial infection or any kind of food intolerance, were excluded). Further differentiation showed that six of 32 patients (19%) in the group of FM and two of 36 patients (6%) in the group of SM had developed chronic diarrhea. The difference was not statistically significant, *p* = 0.14.

## Discussion

Our clinical data correspond to the data from large registry studies, and therefore our patients can be regarded as a representative patient cohort (18, 19). For the first time, we evaluate a weight-adjusted TAC C/D ratio as individual marker of TAC metabolism in order to estimate individual TAC exposure and TAC toxicity in pediatric patients during the first year after LTX in practical routine. Patients with a rapid TAC metabolism rate (FM) after liver transplantation may have a higher risk for a TAC-associated disease compared to those patients who have low metabolism rate (SM).

### TAC C/D Ratio, Patients' Age, and Anthropometric Data

In our study, FM of TAC were noticeably younger than SM. Matching this result, the other anthropometric parameters (body height, body weight, body surface area, and body mass index) were significantly lower in the group of FM compared to SM. The observation that children need higher weight-adjusted TAC doses compared to adult patients to achieve similar blood levels has already been described (20). Moreover, it has been shown several times that younger children need higher weight-adjusted TAC doses than older children to have the same blood levels (21–23). This phenomenon could be explained by age-dependent differences in maturation of TAC pharmacokinetics, such as intestinal first-pass metabolism, volume of distribution, protein binding, and/or hepatic metabolism (24). This information implicates that younger children might be more susceptible to the adverse effects of TAC. One study showed that younger children are more susceptible to cyclosporine A-induced nephropathy (25). Similar studies with TAC have not yet been performed.

### TAC C/D Ratio and Primary Disease Leading to LTX

FM had more frequently biliary atresia as their primary disease compared to SM. PFIC was seen more often in the group of SM. One most rational explanation for these differences could be the difference in patients' age at the time of LTX in these two entities. Patients with biliary atresia were markedly younger at the time of LTX than patients with PFIC. Other studies, which would examine the influence of primary liver disease on TAC metabolism rate in children, are not known. The different absorption rates of TAC in the intestinal tract may play a role. In adults, primary biliary cirrhosis was a more common diagnosis in SM, while autoimmune hepatitis was more frequent in FM (15).

### Tacrolimus C/D Ratio and Renal Function

Previous studies examining the association between TAC metabolism and renal function used various formulas (including chronic kidney disease epidemiology collaboration equation, modification of diet in renal disease equation, and creatinine-based Cockcroft–Gault formula) to estimate renal function in adults (11, 14, 15). It is known that in the first year of life kidney function approaches the normal range in childhood. As the renal function was calculated using creatinine-based Schwartz formula, we saw no significant difference between the groups, as the change of renal function during the first year was calculated. Moreover, renal function increased slightly in both groups in the first year after LTX. Nephrotoxicity was not a considerable clinical concern in our cohort. Overall, we could not prove that FM are more prone to nephrotoxicity than SM, in contrast to previous observations in adult studies (14, 15, 26).

### TAC C/D Ratio and Liver Function

Single case reports mention TAC-induced elevation of transaminases and cholestasis after solid organ transplantation (27–29). In one report, six children developed cholestasis after steroid refractory transplant rejection. Cholestasis disappeared after therapy with TAC was withdrawn (30). However, hepatotoxicity is not considered to be a common side effect of TAC (31). Our study showed no evidence that FM or SM could cause hepatotoxic effects. Because TAC is mostly metabolized in the liver, liver graft function influences TAC pharmacokinetics (32). Disturbed liver function can reduce TAC clearance by two-thirds and increases its half-life three times (33). No ideal laboratory marker has been defined to estimate metabolic liver capacity. Lidocaine metabolism to test liver function is measured by monoethylglycinexylidide test (34). Lidocaine is metabolized primarily by the liver cytochrome P450 system. Serial examinations of liver graft recipients were performed (35, 36). An elevated serum bilirubin could be a sign for cholestasis and could be related to biliary excretion of TAC. A positive correlation between serum bilirubin concentration and TAC trough level has been demonstrated several times (33, 37, 38).

### TAC C/D Ratio and EBV Infection and PTLD

EBV infection is a common complication after pediatric LTX. Almost 20% of pediatric patients develop symptomatic EBV infection after LTX (39). Furthermore, 6% of patients show clinical manifestation of EBV infection in the first year after LTX (40). Another well-known complication after pediatric LTX is PTLD (41). Therapy with TAC and EBV infection are among the main risk factors for the development of PTLD (42). The risk of PTLD after pediatric LTX is estimated to be 5–15%. The largest number of new PTLD cases is diagnosed in the first year after liver transplantation (41, 43). Lower-dose immunosuppressive regimes reduce the incidence of PTLD (44). The role of individual TAC metabolism rate on the development of PTLD has not yet been examined. Our study shows that FM have a significantly higher risk of EBV infection after LTX. The incidence of PTLD in the present study was similar to those in previous studies. All patients developed PTLD in the gastrointestinal tract, were very young, FM, and had hypogammaglobulinemia. All three children with PTLD had an extremely low TAC C/D ratio and needed high absolute TAC doses (mg/day), which therefore increased intestinal exposure. In FM, the TAC level may underestimate the AUC, leaving children over-suppressed and therefore at increased PTLD risk. This observation shows that the calculation of TAC C/D ratio could be helpful to predict the risks of EBV infection and PTLD.

### TAC C/D Ratio and Diarrhea

There is a well-known observation that, with diarrhea, TAC trough levels increase in the blood. On the other hand, diarrhea could also be an adverse effect of TAC. Up to 22–72% of patients on TAC treatment develop diarrhea at some point (33). In the present study, diarrhea was documented in 11.8% of all patients after exclusion of other obvious reasons for diarrhea (e.g., gastrointestinal infection, PTLD, or food intolerance).

### Study Limitations, Clinical Implications, and Further Studies

Our study is limited by the varying quality of data documentation as well as the heterogeneity and small number of patients. There is no control group, and detailed pharmacokinetic determination is missing. Because of the retrospective single-center design for data collection, documentation gaps could not be avoided. Interaction with co-existing diseases and co-medication (e.g., MMF) could not be analyzed in detail. Creatinine-based Schwartz clearance likely overestimated the eGFR for children after LTX.

Previous studies proposed to calculate TAC C/D ratio at different points of time after LTX: two studies calculated the ratio at only 6 months after LTX, and one study calculated the average C/D ratio out of three measurements within the first 6 months after LTX (11, 14, 15). The individual TAC metabolism in children changes over time, even over the years after organ transplantation (33). Therefore, the estimation of TAC C/D ratio in a pediatric patient cohort should be a continuous repetitive process, which allows to help to assess and reassess the individual risk for therapy complications during the follow-up period. Children who continuously show fast TAC metabolism [C/D ratio <51.83 ng/ml/(mg/kg)] should be identified as high-risk patients and should be monitored with a high level of suspicion for possible therapy-related complications (especially EBV infection, PTLD, and TAC nephropathy). Early individual preventive actions, such as reduction of TAC target blood levels or change of immunosuppression, should be considered if early signs of therapy complications develop (e.g., rising EBV DNA load in blood or worsening renal function). Our proposed cutoff value of 51.83 ng/ml/(mg/kg) resulted from four single calculations of C/D ratio at different points of time. Validation of this value and calculation are necessary in further studies with pediatric patients.

## Conclusions

TAC is an effective and well-tolerated drug after pediatric LTX. The calculation of individual, weight-adjusted TAC C/D ratio after LTX in pediatric patients may be a helpful tool for physicians in the daily routine to estimate the risk of therapy-associated complications, such as EBV infection, and to make individual preventive adjustments. In FM, lower levels of TAC are tolerable, especially in the presence of EBV infection, worsening renal function, or in the first 2 years of life. We also found some evidence of other implications and further studies with larger numbers of patients might show additional benefits of this method. The TAC C/D ratio may be usable for a personalized transplant medicine in the future.

## Data Availability Statement

The raw data supporting the conclusions of this article will be made available by the authors, without undue reservation.

## Ethics Statement

The studies involving human participants were reviewed and approved by ethic committee of University Duisburg-Essen. Written informed consent from the participants' legal guardian/next of kin was not required to participate in this study in accordance with the national legislation and the institutional requirements.

## Author Contributions

BP and EL contributed to the study conception, design, and written the first draft of the manuscript. BP performed the data collection, interpretation of results, and statistical analyses. All the co-authors reviewed and approved the final manuscript.

## Conflict of Interest

The authors declare that the research was conducted in the absence of any commercial or financial relationships that could be construed as a potential conflict of interest.

## Abbreviations

Alb: albumin in serum
ALT: alanine aminotransferase
AUC: area under the curve
BDA: biliodigestive anastomosis
bili: total serum bilirubin
BMI: body mass index
BSA: body surface area
CKD-EPI: chronic kidney disease epidemiology collaboration
CNI: calcineurin inhibitor
C/D ratio: concentration/dose ratio
DD: deceased donor
DNA: deoxyribonucleic acid
EBV: Epstein–Barr virus
eGFR: estimated glomerular filtration rate
FM: fast metabolizers
IgG: immunoglobulin G
LD: living donor
LTX: liver transplantation
MDRD: modification of diet in renal disease
MEGX: monoethylglycinexylidide
Mg: magnesium in serum
MMF: mycophenolate mofetil
PCR: polymerase chain reaction
PFIC: progressive familial intrahepatic cholestasis
PTLD: post-transplant lymphoproliferative disorder
RBC: red blood cell count
SM: slow metabolizers
TAC: tacrolimus
TDM: therapeutic drug monitoring
Urea: urea.
